# Pathological changes in the spleen of mice subjected to different time courses of restraint stress

**DOI:** 10.1038/s41598-024-64475-w

**Published:** 2024-06-12

**Authors:** Lei Lei, Yingmin Li, Meili Li, Hongjian Xin, Xiaofei Tian, Yifan Zhang, Weibo Shi, Bin Cong

**Affiliations:** Hebei Key Laboratory of Forensic Medicine, Collaborative Innovation Center of Forensic Medical Molecular Identification, College of Forensic Medicine, Hebei Medical University, No.361 Zhongshan Dong Road, Shijiazhuang, 050017 China

**Keywords:** Immunological techniques, Microscopy

## Abstract

The objective of this study was to investigate spleen pathology and immune cell subset alterations in mice exposed to acute and chronic restraint stress over various timeframes. A deeper understanding of stress-induced spleen injuries can provide new insights into the mechanisms underlying stress-induced disorders. C57BL/6N mice were restrained for different durations (1, 3, 7, 14 and 21 days) for 6–8 h daily. The control mice were observed at the same time points. Post restraint, behavioural experiments were conducted to assess spleen weight, gross morphology and microscopic histological changes. Immunohistochemical staining was used to detect changes in glucocorticoid receptor (GR) expression, immune cell subsets and cell proliferation in response to stress. Our analysis revealed significant behavioural abnormalities in the stressed mice. In particular, there was an increase in the nuclear expression of GR beginning on Day 3, and it peaked on Day 14. The spleens of stressed mice displayed a reduction in size, disordered internal tissue structure and reduced cell proliferation. NK cells and M2-type macrophages exhibited immune cell subset alterations under stress, whereas T or B cells remained unaltered. Restraint stress can lead to pathomorphological alterations in spleen morphology, cell proliferation and immune cell counts in mice. These findings suggest that stress-induced pathological changes can disrupt immune regulation during stress.

## Introduction

Stress is the body’s nonspecific systemic response to cope with the physiological demands caused by various factors that disrupt homeostasis. However, when stress surpasses manageable thresholds, it can exert deleterious effects on the physiological well-being of an individual. Currently, stress-induced injury has been identified as a crucial area of research in both domestic and international contexts due to its association with health disorders and economic burden^1^. Nevertheless, the molecular mechanisms underlying stress-induced damage remain inadequately elucidated. Our team’s previous research on the molecular mechanisms underlying stress-induced injury to the central nervous system revealed a close association between pathological damage and multiple signalling pathways^2–5^. For instance, the impact of GABAA receptors in the dorsomedial hypothalamic nucleus on cardiac function in rats has been investigated, as well as the pathological myocardial cell injury mediated by mitochondrial fission and endoplasmic reticulum stress-related pathways in rats^6,7^. Furthermore, our recent research has led to a series of comprehensive studies focusing on the impact of stress on distal vital organs. Our research findings strongly suggest that stress plays a significant role in various physiological processes. Notably, chronic and prolonged exposure to stress has been found to promote lipid oxidation, influence lipid distribution in atherosclerosis and increase susceptibility to arterial thrombosis^8^, which can lead to cardiovascular and cerebrovascular diseases. Adverse psychosocial conditions resulting from stress have been identified as being a significant contributing factor in the pathogenesis of inflammatory bowel disease^9^. It has also been hypothesised that stress may promote breast epithelial cell carcinogenesis and accelerate breast cancer metastasis^10^. Overall, stress has been implicated in the pathogenesis of numerous immune-mediated chronic diseases, including cardiovascular disease, diabetes, multiple sclerosis, Alzheimer’s disease and various malignancies. In these diseases, stress-induced alterations in immune function and proinflammatory and anti-inflammatory cytokine production, as well as perturbations in various hormones, growth factors and microorganisms, play critical roles^11,12^. The global experience during the novel coronavirus epidemic, which involved infection fears, forced remote work and anxiety stemming from reduced income, has significantly impacted human physical and mental health. These stressors can impair the functions of immune organs and increase susceptibility to other diseases. However, despite acknowledging the substantial impacts of stress on the immune system, comprehensive research in this domain is still in its nascent stages, thus necessitating extensive foundational investigations. As the largest immune organ and a key hub in the peripheral nervous system, the spleen plays a vital role in the body’s immune response^13^. Any external injurious factor can induce changes in the spleen. Therefore, this study aimed to systematically observe pathological changes in the spleen under stress and the effects of stress on immune cell morphology. Based on the successful establishment of a stress animal model, this study aimed to provide a morphological foundation for subsequent in-depth research, which is of immense significance for detailed mechanistic studies in the future.

## Results

### Restraint stress induced increased GR expression and abnormal mental behaviour in mice

**Figure 1:**
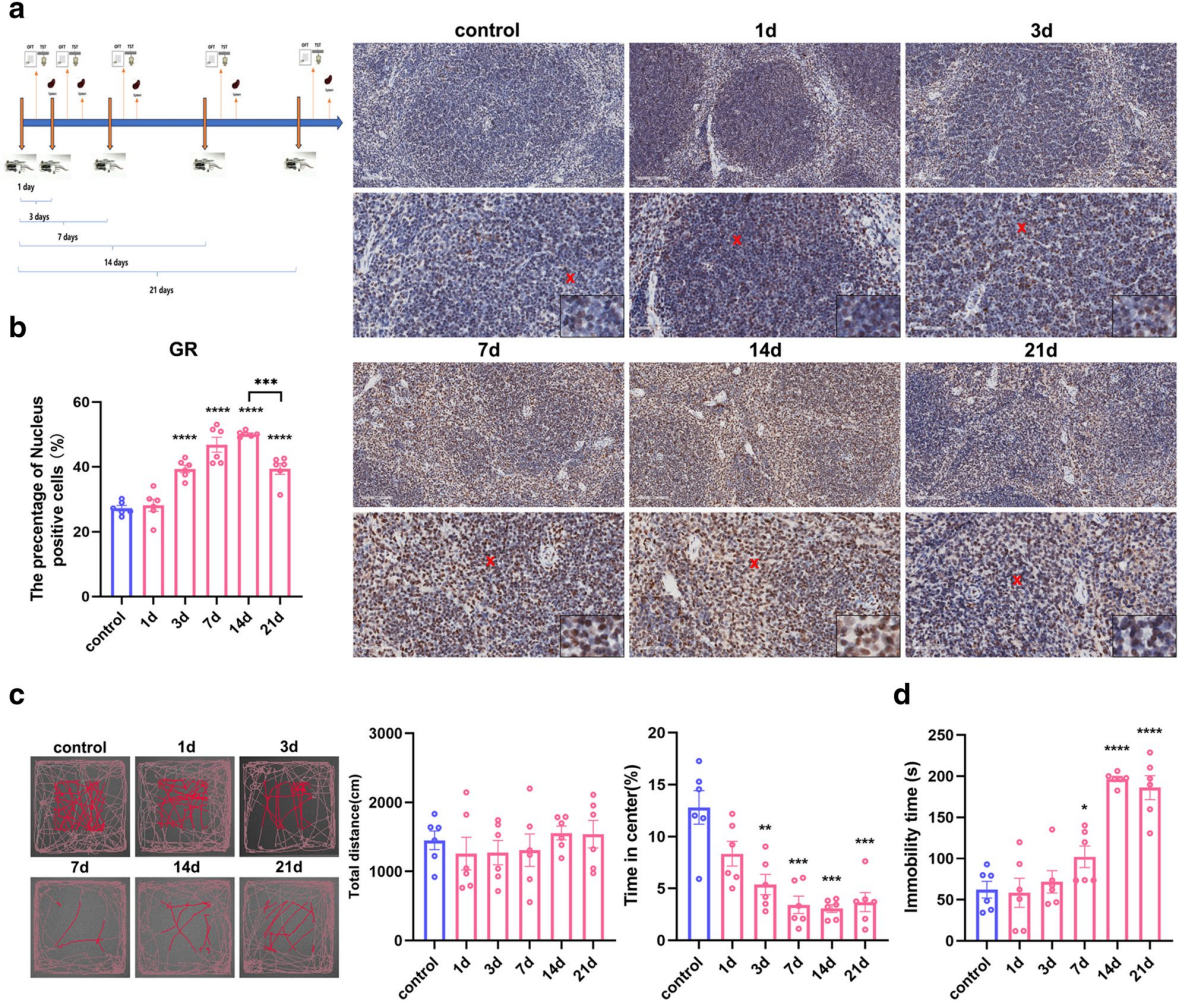
GR increased expression and anxiety-depression-like behavioural performance after restraint stress. (**a**). Different time courses of restraint were performed by using mouse restraint tubes (the positions of the thick orange arrows indicate the time periods when the mice were stressed for long periods of time, the first arrow indicates 1 day; and the thin orange arrows indicate Day 1 and 2 after the thick orange arrows). (**b**). GR immunohistochemical nuclear expression increased after stress for 3 days, peaked after 14 days and decreased after 21 days (20x & 40x, scale bar = 100 μm & 50 μm). (**c**). No significant difference in the trajectory or total distance moved was observed in the open field test, and the central area movement time decreased after 3 days of stress. (**d**). Increased duration of chronic stress at rest in tail suspension experiments. (The position of the "**x**" is the position of the partially enlarged image in the lower right corner, scale bar = 12.5 μm; this scale is true for all of the figures below). Values are expressed as the mean ± SEM, n = 6, *P < 0.05, **P < 0.01, ***P < 0.001, ****P < 0.0001 vs. the control group. ***P < 0.001, 21-day group vs. 14-day group.

The restraint stress model was carefully established as described in Fig. 1a. GR binds to GCs and migrates into the cell nucleus. Therefore, nuclear positivity was used as a marker for immunohistochemical analysis. There was no significant change in the number of mice exposed to restraint stress for 1 day. However, after 3 days of stress, a significant increase in the percentage of GR-positive nuclei was observed, peaking at 14 days post stress (F_0.05(5,30)_ = 38.84, P < 0.0001). However, after 21 days of stress, there was a decrease in positivity compared to 14 days (F_0.05(5,5)_ = 24.34, P < 0.001) (Fig. 1b). This indicated the initiation of GC secretion at 3 days of stress, thus validating the successful construction of the model. The subsequent open field test revealed that there was no difference in the total distance moved by mice across different stress durations compared to the control. However, there was a significant reduction in the proportion of time spent by mice in the central region after stress for 3 days (F_0.05(5,30)_ = 13.11, P < 0.1, P < 0.001) (Fig. 1c), thus suggesting the occurrence of anxiety-like behaviour post restraint stress. After an interval of 6 h, tail suspension experiments were performed. The findings demonstrated a significant increase in quiescence duration during the 7-day, 14-day, and 21-day periods of chronic stress (F_0.05(5,30)_ = 23.88, P < 0.1, P < 0.0001) (Fig. 1d), thus indicating the development of depression-like behaviour as the stress persisted. These findings underscore the successful construction of the restraint stress model and highlight the multifaceted impact of stress on both physiological and psychological aspects.

### Pathological changes in gross and microscopic morphology in mice after restraint stress

**Figure 2:**
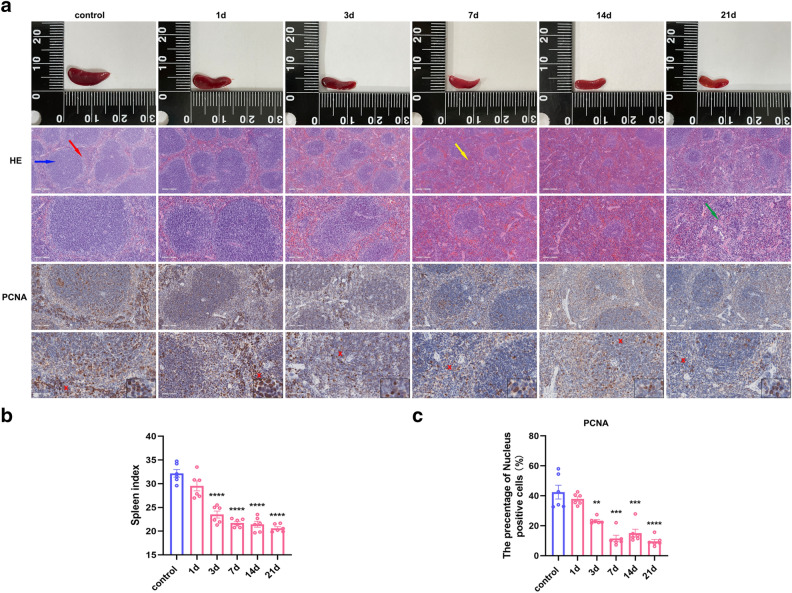
Reduced spleen size, disorganisation of internal tissue structure and reduced cell proliferation in mice after restraint stress. (**a**). Gross and microscopic morphological changes (10x & 20x, scale bar = 200 μm & 100 μm.) The blue arrow indicates white pulp. The red arrow indicates the red pulp. The yellow arrow indicates congestion of the red pulp. The green arrow indicates vascular wall oedema. (**b**). The spleen index decreased significantly after 3 d of stress. a&c. The percentage of positive nuclei and percentage of positive nuclei in the PCNA immunohistochemistry experiments significantly decreased after 3 days of stress (20x & 40x, scale bar = 100 μm and 50 μm). The values are expressed as the means ± SEMs; n = 6; **P < 0.01, ***P < 0.001, ****P < 0.0001 vs. the control group.

Upon dissection of the spleen after restraint-induced stress, the spleen size of the mice was found to have decreased, especially after 3 days of stress (Fig. 2a). The spleen index was calculated as the spleen mass (mg)/mouse body mass (g) × 10. Similarly, the spleen indices of the mice also significantly decreased after stress for 3 days (F = 47.98, P < 0.0001) (Fig. 2b), which is consistent with the gross changes in the mice. Proliferating cell nuclear antigen (PCNA) serves as an indicator of cell proliferation. The immunohistochemical results showed that the percentage of PCNA nuclear-positive spleen cells was significantly lower after stress for 3 days (F_0.05(5,30)_ = 30.70, P < 0.01, P < 0.001, P < 0.0001), thus indicating decreased cell proliferation and consequently affecting spleen size and function (Fig. 2c). H&E staining that after 1 day of stress, the morphological changes in the spleen were small; however, after 3 days of stress, the splenic corpuscle shrank, became disorganised, or even disappeared. Moreover, the congestion of red pulp was significantly aggravated with increasing stress, and pathological changes, such as central artery wall oedema (which is particularly prominent under chronic stress), were observed in some splenic corpuscles.

### Effect of restraint stress on splenic NK cells in mice

**Figure 3:**
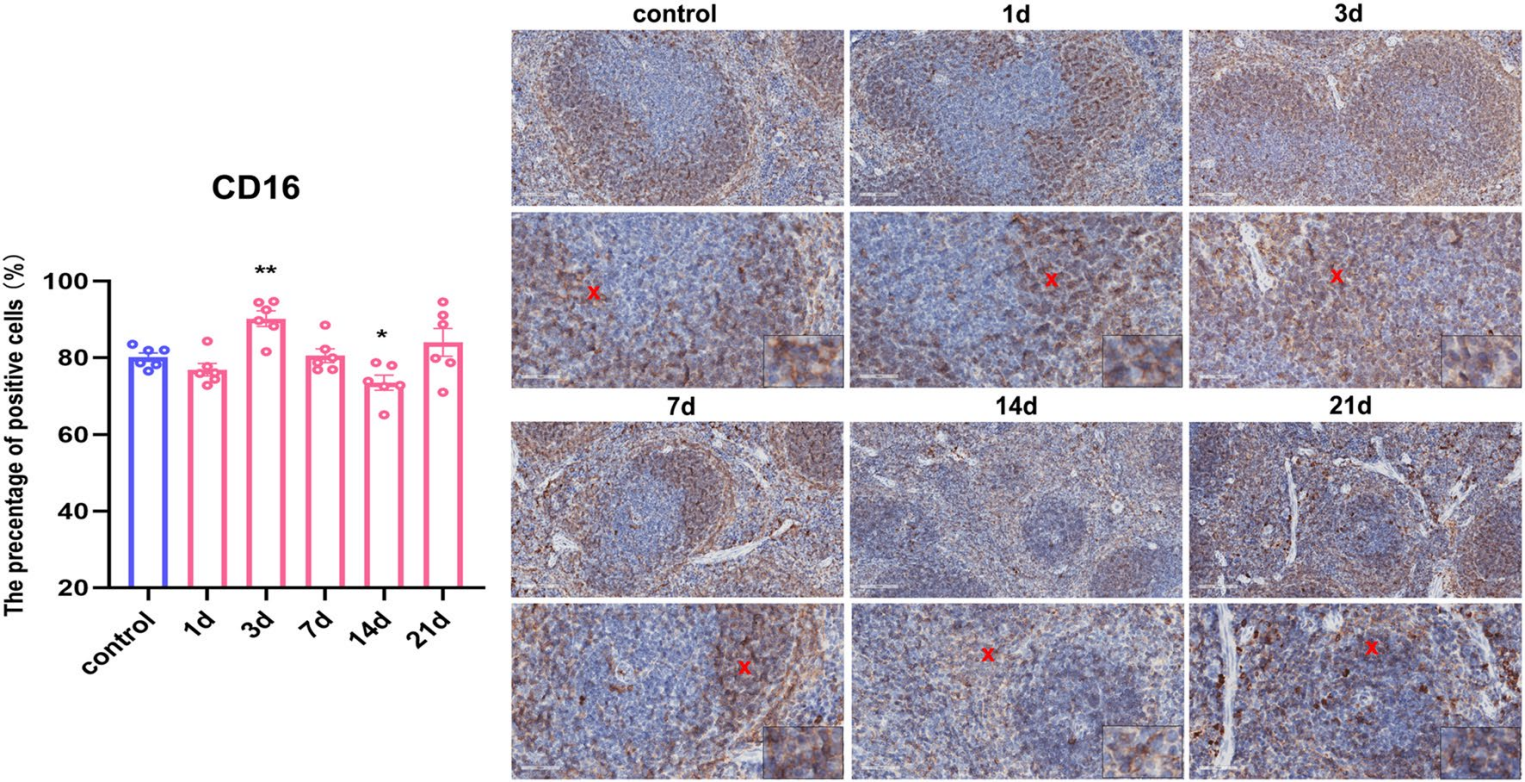
The percentage of splenic NK cells increased after 3 days of restraint stress and decreased after 14 days. Immunohistochemical membrane-positive expression of CD16^+^ on NK cells (20x & 40x, scale bar = 100 μm and 50 μm) and the percentage of membrane-positive cells increased after 3 days of stress and decreased after 14 days of stress. Values are expressed as the mean ± SEM, n = 6, *P < 0.05, **P < 0.01 vs. the control group.

The corresponding specific antibodies, such as cell markers, were used to stain and label each type of immune cell subset in the spleen via immunohistochemical experiments, and the following markers were expressed on the cell membrane; thus, the percentage of membrane-positive cells was statistically determined. There was a significantly greater percentage of CD16-positive NK cells in the stressed group at 3 days than in the control group, followed by a decrease at 14 days of stress (F_0.05(5,30)_ = 7.086, P < 0.05, P < 0.01) (Fig. 3).

### Effects of restraint stress on splenic macrophages and their subtypes in mice

**Figure 4:**
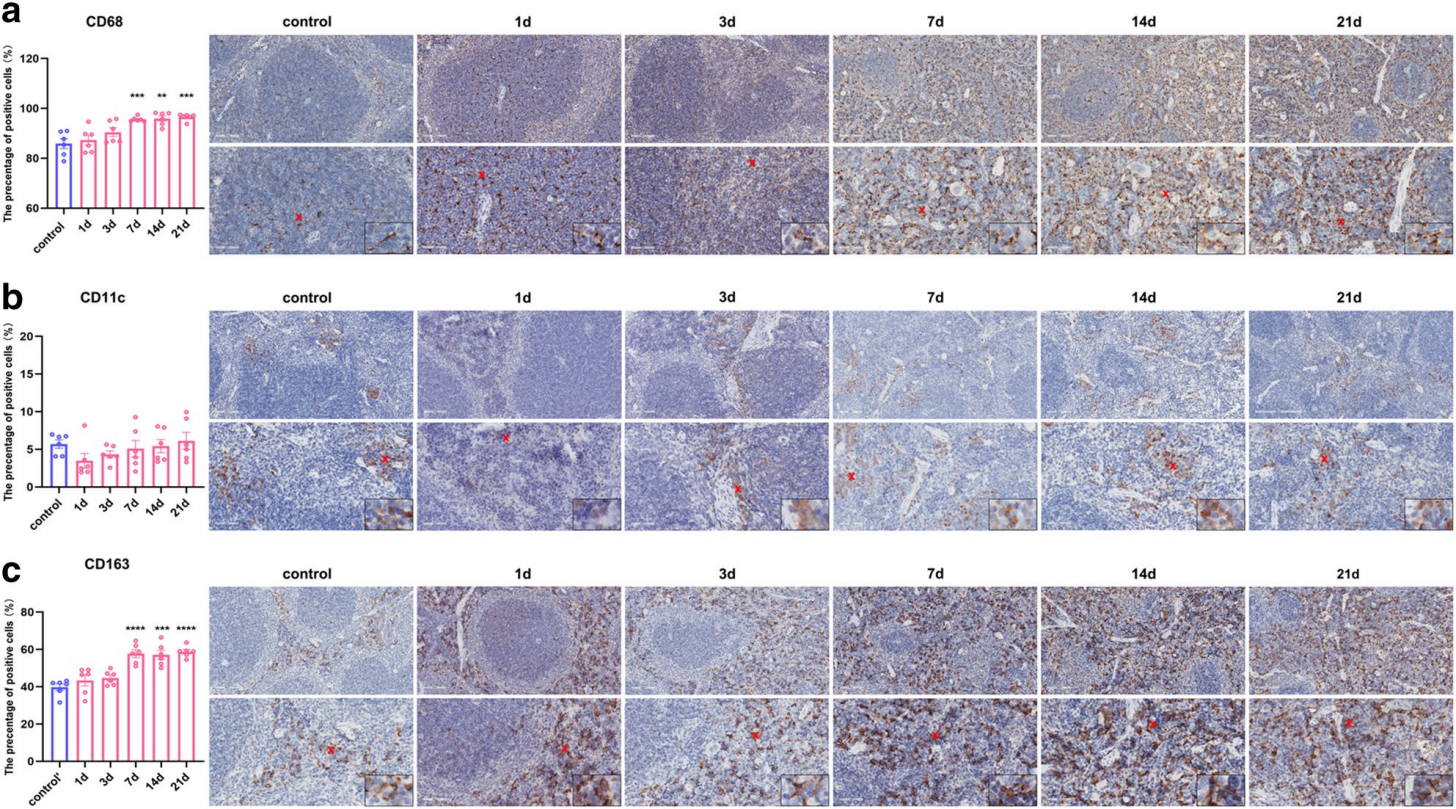
The number of spleen M2 macrophages increased after chronic restraint stress (**a**). Immunohistochemical membrane-positive expression of macrophages labelled with CD68^+^CRS. (**a**). The percentage of CD68-positive macrophages on the immunohistochemical membrane (20x & 40x, scale bar = 100 μm & 50 μm) and the percentage of M2 macrophages on the membrane increased after 7 days of CRS. (**b**). There was no significant difference in immunohistochemical membrane-positive expression (20x & 40x, scale bar = 100 μm & 50 μm) or the percentage of CD11c^+^M1 macrophages that were membrane positive. (**c**). Immunohistochemical membrane-positive CD163^+^ M2 macrophages (20x & 40x, scale bar = 100 μm & 50 μm) and the percentage of membrane-positive cells increased during 7 days of chronic stress. The values are expressed as the means ± SEMs; n = 6; **P < 0.01, ***P < 0.001, ****P < 0.0001 vs. the control group.

In addition to lymphocytes, macrophages, which are pivotal in the spleen, exhibited significant changes under stress. Immunohistochemical labelling of macrophages with CD68 antibodies revealed that the percentage of macrophage membrane-positive cells significantly increased after 7 days of chronic stress (F_0.05(5,30)_ = 10.40, P < 0.01, P < 0.001) (Fig. 4 a). Further analysis of M1 macrophages and M2 macrophages revealed no significant changes in CD11c^+^M1 macrophages (Fig. 4b), whereas CD163^+^M2 macrophages exhibited a significant increase (F_0.05(5,30)_ = 16.87, P < 0.001, P < 0.0001), thus indicating a predominance of M2 macrophages among the increased macrophage numbers (Fig. 4c).

### Effects of restraint stress on T and B cells and their subtypes in the mouse spleen

**Figure 5:**
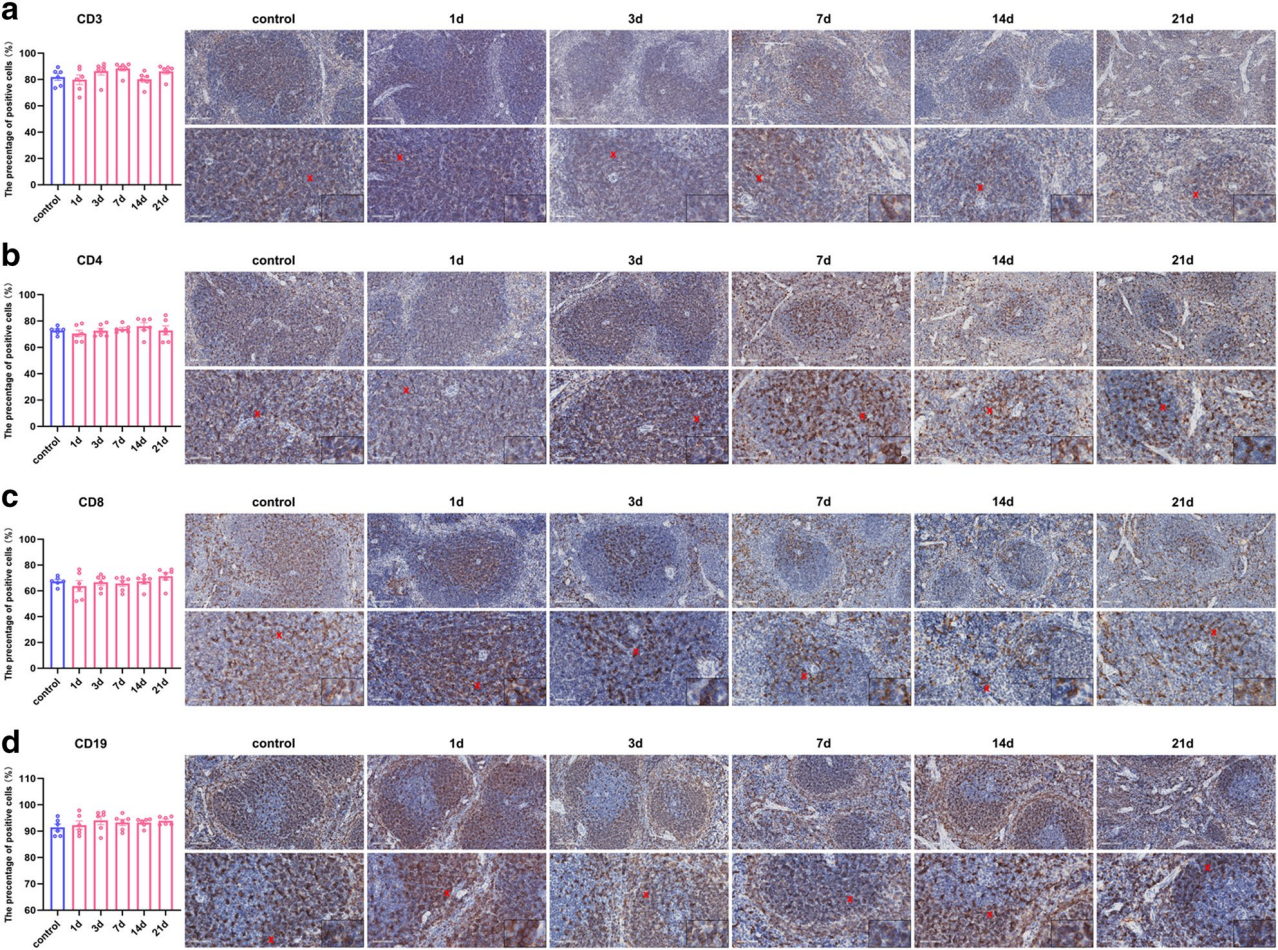
Splenic T and B lymphocytes were not affected by restraint stress. (**a**). There was no significant difference in immunohistochemical membrane-positive expression (20x & 40x, scale bar = 100 μm & 50 μm) or the percentage of CD3^+^-labelled mature T lymphocytes. (**b**). There was no significant difference in immunohistochemical membrane-positive expression (20x & 40x, scale bar = 100 μm & 50 μm) or the percentage of membrane-positive helper T lymphocytes labelled with CD4^+^. (**c**). There was no significant difference in immunohistochemical membrane-positive expression (20x & 40x, scale bar = 100 μm & 50 μm) or the percentage of membrane-positive cytotoxic suppressor T lymphocytes labelled with CD8^+^. (**d**). There was no significant difference in immunohistochemical membrane-positive expression (20x & 40x, scale bar = 100 μm & 50 μm) or percentage of membrane-positive expression in CD19^+^ B lymphocytes. The values are expressed as the means ± SEMs, n = 6.

Immunohistochemical assessment of CD3^+^ mature T lymphocytes, CD4^+^ helper T lymphocytes, CD19^+^ B lymphocytes and CD8^+^ cytotoxic suppressor T lymphocytes no significant changes in the postrestraint stress group compared to the control group (Fig. 5a–d).

## Discussion

The impact of both physical and mental stress on GC levels has been extensively documented and is primarily attributed to the activation of the hypothalamic‒pituitary‒adrenal (HPA) axis^14^. GR translocates from the cytoplasm to the nucleus, where it binds to its ligand GR, thus serving as a key indicator of stress and playing a pivotal regulatory role along with related transcription factors^1,15,16^. In this study, we observed dynamic changes in the percentage of GR-positive nuclei at different stress time points during stress exposure, with a peak being observed on Day 14. This finding provides evidence for the successful establishment of our mouse model of restraint stress. Notably, the decrease in GR nuclear positivity that was observed on Day 21 suggested potential adaptability of the mice to restraint stress.

This study is consistent with previous clinical observations, thus highlighting the profound impact of prolonged immobilisation on both the physical and mental well-being of an individual^17^. For example, in the case of body weight changes in mice after stress, other members of our group performed detailed monitoring under the exact same conditions of mice and modelling methods. The researchers found that the rate of body weight gain was reduced after stress. In this study, mice exposed to acute and chronic restraint stress exhibited varying degrees of depression- and anxiety-like behaviours^18^. Concomitantly, the spleen tissues displayed pathomorphological changes, including volume reduction, spleen index reduction, abnormal tissue structure and diminished cell proliferation. Therefore, we hypothesised that the reduced proliferative properties of splenocytes after stress may be the main reason for the reduction in spleen size and the decrease in the splenic index. These morphological alterations in the spleen reflect stress-induced pathological damage, whereas the systemic response to stress influences modifications in immune cells. As a key component of the innate immune system, NK cells protect the body from bacterial and viral infections and malignancies^19^. Due to the ability of NK cells to elicit immune responses without prior sensitisation^20^, the activation receptor cluster of NK cells increases during restraint stress, thereby promoting heightened cellular activity of NK cells^21^. This characteristic endows NK cells with the ability to respond quickly and accurately when the body is subjected to acute stress stimuli. Our experimental results confirmed that the percentage of NK cells significantly increased at 3 days of acute stress, thus indicating that acute stress stimuli promoted NK cell proliferation and activation of the immune response and thereby playing an important role in resisting adverse external stimuli and protecting cells. However, the increase in NK cells was not sustained with increasing stress duration. After 14 days of stress exposure, GC levels peaked, and the percentage of positive NK cells significantly decreased, which was possibly due to the resistance of NK cells to excessive endogenous GCs caused by stress^22^, which compromised their cellular protective capacity.

Resident tissue macrophages, which are abundantly distributed across various organ systems, are present in unusually high numbers and primarily originate from monocyte recruitment^23^. Chronic stress induces immune suppression in the spleen, thereby increasing vulnerability to adverse stimuli. Consequently, there is a heightened need for enhanced monocyte recruitment and subsequent monocyte differentiation into macrophages. Macrophages located in the marginal zone of white pulp can rapidly recognise antigens. Furthermore, these macrophages play a crucial role in phagocytosing pathogens that enter the systemic circulation of the spleen while also serving as antigen-presenting cells^24^.

Stress-induced structural disorders of spleen tissue and increased apoptosis of splenocytes require heightened phagocytic activity to remove cell debris and broken red blood cells. The process of macrophage differentiation into distinct phenotypes upon activation is known as polarisation. During this process, macrophages can polarise into the M1 and M2 subtypes in response to different stimuli^25^. However, Fleur S et al. reported that M2 macrophages have a stronger phagocytic ability than M1 macrophages^26^. Similarly, in this study, the percentage of M2 macrophages that were membrane positive was significantly increased during chronic stress, whereas there was no significant difference in the percentage of M1-type macrophages. This increase in M2 macrophages suggested that stress could cause potential pathological damage to spleen cells, thus necessitating a stronger phagocytic response from M2-type cells. Additionally, it has been reported that GC-induced tumour necrosis factor promotes phagocytosis in macrophages^27^. Furthermore, GCs can also promote M2c polarisation by attenuating the expression of proinflammatory genes in macrophages, thereby inhibiting their polarisation towards M1^28,29^. Therefore, we hypothesise that the stress-induced elevation in GCs could contribute to the increase in splenic macrophages and their polarisation towards the M2 phenotype. Furthermore, HE staining revealed severe congestion in the red pulp of the spleen and an unclear boundary between the white pulp and red pulp after stress exposure. Previous studies have confirmed that when M2 macrophages are activated^30^, their primary role is to eliminate foreign bodies, such as cell debris, and promote the repair and remodelling of blood vessels and histiocytes. These macrophages also release a significant quantity of anti-inflammatory cytokines, including IL-10 and IL-13, which enter the bloodstream via the splenic sinusoids^31^. These cytokines have anti-inflammatory effects not only in the spleen but also in other distant organ tissues. Furthermore, a notable number of monocytes were observed in the red pulp of the spleen, thus indicating their potential to exit the spleen and migrate to other target tissues, such as tumours^32^. It is also worth noting that recruited macrophages in these spleens play a crucial role in the tumour microenvironment once they enter tumour tissues^33,34^. Given these specific functions of M2 macrophages and the observed increase in their population under stress, it is important to conduct further explorations to better understand the molecular mechanisms underlying how M2 macrophage proliferation affects the tumour microenvironment during stress. This new line of inquiry could lead to valuable insights into the impact of stress on immune function and tumour development.

This was an observational and descriptive study aimed at the preliminary observation of the effects of different durations of stress on splenic cell subpopulations. The changes of spleen under stress can be more clearly and directly observed through immunohistochemical experiment labelling of cell subsets. However, some methodological shortcomings, such as the identification of some cell subpopulations, such as NK cells, is limited due to the lack of double-labelling immunohistochemical staining experiments.

## Conclusion

Our study highlights the significant impact of restraint stress on spleen health and highlights notable changes in spleen morphology and function during acute stress. These changes include a reduction in spleen size, disrupted histomorphology, reduced proliferation capacity, and an increase in NK cell numbers. In contrast, chronic stress resulted in a decrease in NK cell numbers and an increase in M2 macrophages, thus suggesting stress-induced splenic injury. These findings provide valuable insights into the mechanisms underlying the interaction between psychological stress and immune function, thus offering new perspectives for the development of novel prevention and treatment strategies for stress-related diseases.

## Materials and Methods

### Tissue

Male C57BL/6N mice aged 7–8 weeks were procured from Beijing Vital River Corporation. After one week of acclimation, the mice were placed in polycarbonate restraint tubes. Ten mice per group were restrained for varying durations (1, 3, 7, 14 and 21 days) for 6–8 h to establish acute and chronic stress models with different time courses. A control group was established in each group for an equal period of time; while the experimental group was restrained, the control group was fasted from food and water and free to move around for the same amount of time. The control group was experimentally evaluated at the same time as the stress group. All of the procedures were strictly based on the ARRIVE guidelines (PLoS Bio 8(6), e1000412,2010​) and were approved by the Institutional Review Board for Animal Experiments at Hebei Medical University (IACUC-Hebmu-2023011). All methods were performed in accordance with the relevant guidelines and regulations, as confirmed. To collect the spleens, the mice were anaesthetised with 2% pentobarbital sodium, and the spleens were carefully excised from the right lower quadrant. The spleens were weighed, photographed, and fixed in 4% neutral formaldehyde. They were then dehydrated by using graded ethanol, cleared and embedded in paraffin wax. The spleens were serially sectioned at 4 μm for further analysis.

### Method

- 1. Mice were placed in the behavioural laboratory 30 min before the experiment. The utilized active test box was composed of 4 rectangular white opaque boxes of 40 cm × 40 cm to avoid excessive light. The mice were placed in the centre of the test box, and the central area was 20 cm × 20 cm in the middle. The mice were allowed to move freely for 5 min in a quiet environment, and their activities were monitored by using a camera system. The analysis software for the EthoVision XT 15 Behaviour Analysis System (Noldus, Netherlands, version 15.0.1416) was used. Mouse faeces and urine were removed with toilet paper, and the interior of the open field was sprayed with 75% alcohol and finally wiped clean with toilet paper to prevent the odours between different mice from affecting the results of the subsequent experiments.
- 2. Four bright 20 cm × 20 cm × 35 cm white Plexiglas arenas were used for the experiment. The tail of each mouse was attached to a hook 3 cm from the top with tape 1 cm from the tip of the tail for 5 min. Immobility was defined as the absence of limb or body movement during passive suspension. Behavioural tests were videotaped and analysed by using an EthoVision XT 15 behaviour analysis system (Noldus, Netherlands, version 15.0.1416). Between each test, the arena was cleaned with 75% alcohol.
- 3. HE-stained paraffin sections were deparaffinized with xylene, hydrated with graded ethanol, and subsequently stained with haematoxylin, hydrochloric acid alcohol and eosin. The sections were hydrated with ascending graded ethanol for 2 s each. Xylene was applied as the clearing agent, and neutral gum was used for mounting.
- 4. Immunohistochemical paraffin sections were deparaffinized with xylene and hydrated with graded ethanol, followed by antigen retrieval at high temperature and high pressure by using EDTA antigen retrieval buffer (pH = 9.0, Maxim, Fujian, China) or sodium citrate antigen retrieval solution (pH = 6.0, Biosharp, Guangzhou, China) according to the package instructions for the antibodies. Endogenous peroxidase blocking solution and goat serum blocking solution were added dropwise for 10 min each. This was followed by overnight incubation with specific antibodies, including anti-glucocorticoid receptor (1:2000, ab183127, Abcam, UK), anti-PCNA (1:500, ab92552, Abcam, UK), anti-CD3 (1:150, ab16669, Abcam, UK), anti-CD4 (1:1000, ab183685, Abcam, UK), anti-CD8 (1:2000, ab217344, Abcam, UK), anti-CD19 (1:1000, ab245235, Abcam, UK), anti-CD16 (1:1000, ab203883, Abcam, UK), anti-CD68 (1:100, ab283654, Abcam, UK), anti-CD163 (1:500, ab182422, Abcam, UK), and anti-CD11c (1:100, ab219799, Abcam, UK), at 4 °C. Biotin-labelled IgG polymer and Streptomyces avidin-peroxidase were each incubated at room temperature for 10 min and visualised with DAB stain (Maxim, Fujian, China). Haematoxylin was used for counterstaining, hydrochloric acid alcohol was used for differentiation, and saturated lithium carbonate reversion blue was used for visualisation. The slides were mounted with neutral gum after ethanol dehydration and xylene clearing. After the images were saved, pathological changes were observed by using an Aperio ScanScope CS2 scanner (Leica, Biosystems, Vista, CA, USA) via the analysis software Aperio Imagescope (version 12.4.6), which is a Digital Pathology Analysis System (Leica, Biosystems, Vista, CA, USA, version 102.0.7.5) ^5–7,35–37^. The entire spleen region was selected for analysis, and protein expression was assessed by using the nuclear V9 and membrane V9 algorithms of this software. The results are reported as the percentage of membrane-positive cells or nuclear-positive cells among all of the splenocytes.
- 5. Statistical analysis: SPSS 25.0 statistical software was used for the statistical analysis (IBM SPSS Statistics, Chicago, IL, USA for statistical analysis, version 25). All of the data are presented as the mean ± SEM. The comparison of the means of each group was performed by using one-way ANOVA, the least significant difference (LSD) method was used for comparisons between two groups, and P < 0.05 was considered to indicate a statistically significant difference. GraphPad Prism 8 software (GraphPad Software Inc., CA, USA, version 8.4.3.686) was used for graph generation.

## Data Availability

Some or all of the data that support the findings of this study are available from the corresponding author upon reasonable request.
